# Notes and News

**Published:** 1893-04-01

**Authors:** 


### NOTES AND NEWS.

Collected and Communicated.

We are glad to learn that the University of Oxford has abandoned the proposal to demand the repayment of £500 from the Radcliffe Infirmary, a sum which was originally granted by the University to the Infirmary as a contribution towards the erection of fever wards, now no longer in use, and consequently of no advantage to the University.

As sermons can be culled from stones, so for the inquiring mind knowledge can be gained everywhere, and all things turned to account. We have before us a small trade journal with the uninviting title "Advertising." Turning over the pages of the small paper, we are surprised to find useful and varied information within the sphere indicated by the title, and shown to be a wider field than we had imagined. Some really excellent hints are given, and also useful information. We would recommend hospital secretaries and other institutional authorities who have to enter the advertising field to send for a copy of "Advertising," to be had at Smith's agency, 28, Hutton Street, Fleet Street, E C. We believe it is also procurable at newsagents and bookstalls, price 2d.

At a meeting of the Royal Statistical Society held last week, Dr. Francis Warner read an interesting paper on the physical and mental condition of children. Dr. Warner's deductions are based on observations of fifty thousand children seen in upwards of one hundred schools. These inquiries have resulted in a conclusion that 16 per 1,000 of the children seen deviate from the normal, or in other words are in some way defective. Low nutrition is found mainly associated with defective development. Defects in the cranium seem to be due to rickets in early life. Children in poor law and industrial schools show greater deviation from the normal than others, whilst marked superiority is stated to exist amongst the children of Jewish parents above those of purely British nationality. We agree with Dr. Warner that careful inquiry as to the influence of social conditions, nutriment, exercise, and education upon the young is a matter of the utmost importance, and since there appears to be a decided tendency on the part of the State to become an Alma Mater, it would be well to determine the best conditions and methods to adopt in the rearing of the young of the coming generation.

Montreal is to have another big medical school. The work is to be commenced immediately.

Preparations for cholera in America include a proposed enlargement by the Quarantine Commissioners of Hoffman Island. On this it is proposed to build an hotel with accommodation for 500 cabin passengers, which will be isolated from the present docks. Further accommodation is projected for steerage passengers. The Commissioners also ask for a site on Swinbourne Island for a three-storey hospital building.

An interesting Conference on Child Saving was recently held in Philadelphia. Charity in the city of Philadelphia is largely occupied with this field of work, possessing no less than sixty-two institutions for the benefit of children. Many speakers at the conference were in favour of removal by the State of children from surroundings of neglect and vice. A large number urged the necessity of guarding against the pauperising tendency of shifting respon sibility from the parents to the State.

A charitable institution of a somewhat novel character has just been presented to New York by a wealthy citizen, Mr. G. Stewart Kennedy. It is titled the United Charities Building. The cost of erection was 700,000 dollars, and it has been placed under the management of a Board of Trustees representing many important charities. Numerous as our charities are, and great the need for them, statistics show that in American cities the growth of charity will have to be even greater than with us, if it is to cope with the increasing calls upon it. Crime in America is augmenting faster than wealth, which condition must bring ultimately attendant poverty. Added to this the large amount of needy and incapable foreigners flocking to American shores, tends largely to the formation of a huge pauper population. The establishment of such an institution as this new United Charities Building should afford opportunities for studying the problem of poverty, and assist in its solution.

Those interested in occultism, either as believers or would-be investigators, will regard with triumph or surprise, as the case may be, the recent investigations by men of science in Italy. The object of these investigations was a Neopolitan woman, quite illiterate, and the wife of a carpenter. Those taking part in the researches included the Astronomer at Milan, a German doctor of philosophy, Professor C. Richet, Editor of the "Revue Scientifique," and Professor Lombroso, the celebrated Alienist. The woman in question had from the earliest years been said to possess unaccountable and uncanny powers. She had, however, not exhibited them for many years. For the purposes of the investigation she was induced to lend herself to a series of *séances*, conducted in the ordinary manner, the utmost care being taken that no collusion should be possible. The *séances* took place in an ordinary room, in darkness, gloom, and brilliant light. The manifestations consisted of the lifting of heavy objects into mid-air, and the appearance of unfamiliar features and of unaccountable sensations of touch. The investigators regard these manifestations as totally unaccountable. A well known Italian scientist is recorded to have said that "the easiest and most probable way to account for the phenomena collectively was to call them the work of spirits."

